# A lumped stiffness model of intermuscular and extramuscular myofascial pathways of force transmission

**DOI:** 10.1007/s10237-016-0795-0

**Published:** 2016-05-18

**Authors:** Michel Bernabei, Huub Maas, Jaap H. van Dieën

**Affiliations:** Department of Human Movement Sciences, Vrije Universiteit Amsterdam, MOVE Research Institute Amsterdam, Van der Boechorststraat 9, 1081 Amsterdam, The Netherlands

**Keywords:** Biomechanics, Phenomenological model, Myofascial force transmission, Stiffness, Connective tissues, Soleus, Gastrocnemius, Rat

## Abstract

Mechanical behavior of skeletal muscles is commonly modeled under the assumption of mechanical independence between individual muscles within a muscle group. Epimuscular myofascial force transmission via the connective tissue network surrounding a muscle challenges this assumption as it alters the force distributed to the tendons of individual muscles. This study aimed to derive a lumped estimate of stiffness of the intermuscular and extramuscular connective tissues and to assess changes in such stiffness in response to a manipulation of the interface between adjacent muscles. Based on in situ measurements of force transmission in the rat plantar flexors, before and after resection of their connective tissue network, a nonlinear estimate of epimuscular myofascial stiffness was quantified and included in a multi-muscle model with lumped parameters which allows for force transmission depending on the relative position between the muscles in the group. Such stiffness estimate was assessed for a group with normal intermuscular connective tissues and for a group with increased connectivity, mimicking scar tissue development. The model was able to successfully predict the amount of epimuscular force transmission for different experimental conditions than those used to obtain the model parameters. The proposed nonlinear stiffness estimates of epimuscular pathways could be integrated in larger musculoskeletal models, to provide more accurate predictions of force when effects of mechanical interaction or altered epimuscular connections, e.g. after surgery or injury, are substantial.

## Introduction

It is traditionally thought that skeletal muscles transmit force to their insertions on bones primarily through the myotendinous junction (Tidball 1991). This common understanding has been challenged by the evidence of secondary pathways of force transmission (Huijing 2009; Maas and Sandercock 2010). According to this new perspective, part of the force produced by a muscle can be exerted on neighboring muscles and surrounding extramuscular structures through a continuous network of connective tissues. Such a network extends from the endomysial-perimysial layers (Trotte et al. 1995; Purslow 2010) to the epimysium of muscles and further to the connective tissues between adjacent muscle bellies (intermuscular) and tissues supporting nerves and blood vessels (extramuscular) (Maas and Sandercock 2010; Higham and Biewener 2011). These inter- and extramuscular pathways provide the means for epimuscular myofascial force transmission (Huijing et al. 1998; Maas et al. 2001; Yucesoy et al. 2006), in addition to the parallel myotendinous path to bone. As a consequence, muscles do not necessarily function as independent actuators.

Until recently, multi-muscle models considered skeletal muscles as independent actuators (Woittiez et al. 1985; Zajac and Winters 1990; Jacobs et al. 1996; Raasch et al. 1997; Correa et al. 2011; Lee et al. 2015). Moreover, these models have commonly been parameterized by dissecting muscles free from the structures surrounding them (Close 1964; Rack and Westbury 1969; Burke et al. 1976; Woittiez et al. 1985; Ettema et al. 1990). Therefore, stiffness of inter- or extramuscular pathways is neglected. A first formal description of extramuscular linkages was encountered in a phenomenological Hill-type muscle model to explain an unexpected, and first ever reported, difference between forces measured at both ends of a muscle (Devasahayam and Sandercock 1992). Despite its novelty, the authors considered this model speculative, as it was not quantitatively tested nor was a stiffness estimate for extramuscular linkages reported. More recently, a specific Hill-type model was extended to approximate the gearing between transverse and longitudinal forces on the muscle belly. Although this model was able to predict changes in muscle force produced by external loads exerted on the muscle belly, the muscle preparation and the resulting parametric model of transverse loading used a single muscle isolated from its surrounding (Siebert et al. 2014a, b). In a preliminary attempt to illustrate, although only qualitatively, the effects of extra- and intermuscular connections, a physical model representing connections between rat ankle dorsi-flexors was devised using springs elements of different stiffness and inextensible linking elements (Maas et al. 2001). Finally, finite element (FE) modeling emerged as a promising approach to account for several passive properties of muscle tissue as a continuum. These models allowed to quantitatively assess mechanisms of force transmission and the effects of mechanical interaction between muscle fibers at the extracellular matrix interface on sarcomere length, shear strain and force (Yucesoy et al. 2002; Sharafi and Blemker 2011; Zhang and Gao 2012), and between whole muscles linked by intermuscular elastic structures (Yucesoy et al. 2003). However, the computational load of FE models makes the integration of such multi-muscle systems into whole limb or body musculoskeletal models more difficult. A simpler model accounting for macroscopic muscle behavior seems, at first instance, preferable. Moreover, the complexity of intermuscular connections and macroscopic differences between muscle groups, e.g. moment arm, number of joints spanned, distance between origin-insertion of neighboring muscles, presents an open challenge for computing local stress–strain maps of the synergistic muscle group as a whole. Also, previous studies have shown that the extent of epimuscular myofascial effects can vary between muscle groups (see Table 1 in Huijing 2009) and species (Maas and Sandercock 2008), which requires modeling tools to be adaptable. Despite the fact that a number of studies have described force transmission via epimuscular myofascial linkages, no stiffness values have been assessed directly from experimental data. Note that the stiffness of epimuscular linkages in above described FE models was the result of optimization (Yucesoy et al. 2002; Maas et al. 2003).

We have shown that the ratio of transmitted force between myotendinous and epimuscular (inter- or extramuscular) pathways changes due to altered mechanical properties of connective tissues between muscles (Bernabei et al. 2016). Such changes can be a consequence of muscle injury (Garrett et al. 1988; Kääriäinen et al. 2000; Silder et al. 2010), disease (Booth 2001; Smith et al. 2011), surgical treatment (Almeida-Silveira et al. 2000; Smeulders and Kreulen 2007) or aging (Kovanen et al. 1987; Ramaswamy et al. 2011). To take into account effects of post-operative scar tissue development after tendon transfer of spastic muscles, a secondary non-myotendinous pathway of force transmission was added to an EMG-driven musculoskeletal model of the quadriceps muscles (Andersen 2009). Although this model provides a simple solution to integrate force transmission in pathological conditions, the ratio of force transmitted to the new insertion and via scar tissue to the patella was arbitrarily set, i.e., not determined by the mechanical properties of the post-operative scar tissues.

The aims of this study were (i) to derive a lumped estimate of stiffness of the intermuscular and extramuscular connective tissues and (ii) to assess changes in such stiffness in response to a manipulation of the interface between adjacent muscles. Based on in situ measurements of force transmission in the rat soleus (SO) and lateral gastrocnemius and plantaris complex ($\hbox {LG}+\hbox {PL}$), before and after resection of their connective tissues network, a nonlinear estimate of stiffness was quantified and included in a phenomenological multi-muscle model with lumped parameters. We used this model to predict the ratio of force transmitted via epimuscular myofascial and myotendinous pathways of SO and $\hbox {LG}+\hbox {PL}$ for both normal and enhanced intermuscular connectivity. Finally, the relative contribution of extramuscular pathways of force transmission, i.e., SO and $\hbox {LG}+\hbox {PL}$ neurovascular tracts, with respect to the epimuscular myofascial and myotendinous pathways was assessed.

## Methods

### In situ experiment

#### Animals

Experiments were performed on male Wistar rats ($n=14$, mean ± SD body mass 308.0 ± 11.1 g), which were divided into two groups: the first group had normal connective tissues ($n=7$, normal connectivity group, NO), while the second one was tested after manipulation of the connectivity between SO and $\hbox {LG}+\hbox {PL}$ ($n=7$, tissue-integrating mesh group, TI). All surgical and experimental procedures were approved by the Committee on the Ethics of Animal Experimentation at the VU University Amsterdam and in strict agreement with the guidelines and regulations concerning animal welfare and experimentation set forth by Dutch law.

#### Surgical procedures for intermuscular connectivity manipulation

One to two weeks prior to the in situ measurements, TI group animals were implanted with a tissue-integrating surgical mesh at the muscle belly interface of SO and $\hbox {LG}+\hbox {PL}$. Surgical procedures for the implantation have been previously reported in detail (Bernabei et al. 2016). Briefly, following preparation for aseptic surgery and inhalation anesthesia (2–3 % isoflurane), the lateral side of the posterior crural compartment was accessed by a partial fasciotomy of the surrounding crural fascia, limiting the exposure of the posterior crural muscles to the proximal two-thirds of the SO and LG muscle-tendon units (MTUs). The distal portion of the biceps (accessory head) and posterior crural fascia, covering the distal myotendinous junctions of SO, LG, medial gastrocnemius and PL as well as the Achilles tendon, were left intact. After blunt dissecting the connective tissues between the dorsal side of the SO and the ventral side of $\hbox {LG}+\hbox {PL}$, a tissue-integrating mesh ($n=7$; Premilene$^{\circledR }$ mesh, B. Braun Melsungen AG, Germany) was sutured to the SO muscle belly at the interface of SO and $\hbox {LG}+\hbox {PL}$ muscles. Meticulous dissection prevented damage of the SO neurovascular tract (NV1), which runs centrally between SO and LG muscle bellies. A one-time pre-operative subcutaneous injection of a pain-killer (0.02 mg/kg, Temgesic$^{\circledR }$, Schering-Plough, Maarssen, The Netherlands) was administered to prevent discomfort of the animal following the survival surgery. Additional doses were given 1–2 days after the surgery if signs of pain were noticed.

#### Surgical procedures for in situ measurements

**Fig. 1:**
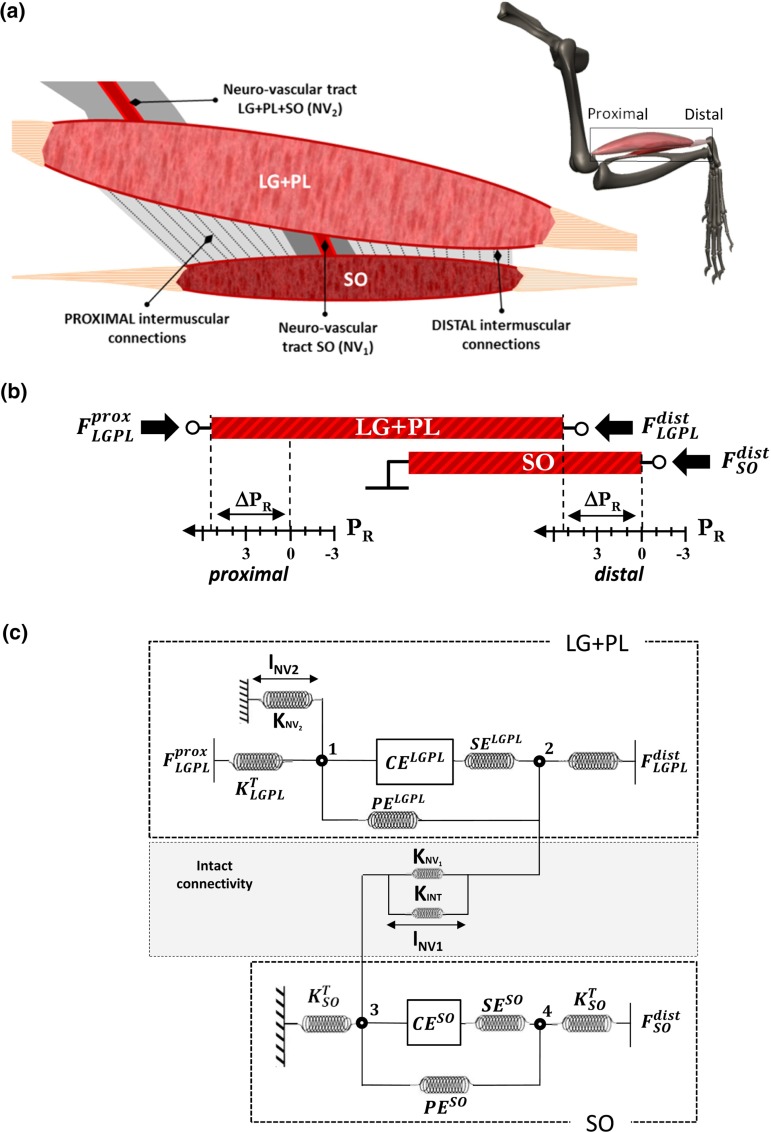
Schematic representation of the experimental setup and of the model. **a** Epimuscular (inter- and extramuscular) pathways which were left intact after dissecting the plantar flexors synergistic group before the in situ measurement. A proximal and a distal intermuscular pathway of force transmission were distinguished, while the connective tissue bundles enwrapping blood vessels and nerves proximal to $\hbox {LG}+\hbox {PL}$ (*NV2*) and at the $\hbox {SO}/\hbox {LG}+\hbox {PL}$ interface (*NV1*) were defined as extramuscular pathways of force transmission. **b** Proximal and distal relative displacement $(P_{R})$ between SO and $\hbox {LG}+\hbox {PL}$ MTUs muscles imposed during the in situ experiment. Tendon forces were measured at the proximal and distal $\hbox {LG}+\hbox {PL}$ tendons as well as at the distal SO tendon. The proximal SO tendon insertion on the tibia was left intact. **c** Lumped-parameter model rendering SO and $\hbox {LG}+\hbox {PL}$ individual actuators with a simple Hill-model composed by a contractile element (CE), both series (SE) and parallel (PE) elastic elements and proximal and distal mass-nodes $(\hbox {m}_{\mathrm{PROX, DIST}})$. The mechanical interaction provided by intermuscular $(\hbox {INT}_{\mathrm{PROX, DIST}})$ and extramuscular (*NV1*, *NV2*) pathways of force transmission were represented with piecewise linear elastic elements between SO and $\hbox {LG}+\hbox {PL}$ lumped-mass elements. $K_{\mathrm{SO}}^{T}, K_{\mathrm{LGPT}}^{T}$: tendon stiffness. $K_\mathrm{NV1}, K_\mathrm{NV2}$: Neuro-vascular tract stiffness (extramuscular), $K_{\mathrm{INT}}$; intermuscular myofascial connective tissues stiffness. $I, \Delta l$: length, length changes in the given element. CE, PE, SE: contractile element, parallel elastic element series elastic element. $\Delta F_{\mathrm{LGPL}}$: proximal-distal $\hbox {LG}+\hbox {PL}$ force difference (*intact*: intact condition; *post I*: myofascial pathway severed; *post II*: intermuscular neurovascular tract severed). $P_{R}$: positions of $\hbox {LG}+\hbox {PL}$ relative to SO muscle, $P_{\mathrm{REF}}$: $P_{R}=0\,\hbox {mm}$. Muscles: reference position corresponding to $90^{\circ }$–$90^{\circ }$ knee-ankle joint angles. $\widehat{{F}_\mathrm{INT}},\widehat{{F}_\mathrm{NV1}}, \widehat{{F}_\mathrm{NV2}}$ :force estimates of non-myotendinous pathways of force transmission, calculated at each different SO/LG+PL relative position $P_{R}$ from experimental data. $\hat{K}_{\mathrm{INT}}$, $\hat{K}_{\mathrm{NV1}}$, $\hat{K}_{\mathrm{NV2}}$: stiffness estimates of non-myotendinous pathways of force transmission; intermuscular $({ INT})$ and extramuscular (${ NV1}, { NV2}$)

According to standard procedures in our laboratory (Maas et al. 2001), the animals were anesthetized by an intraperitoneal injection of urethane solution (1.2 ml/100 g body mass, 12.5 % urethane solution). Extra doses were administered if necessary (1.5 ml maximally) until withdrawal reflexes to a pain stimulus were suppressed completely. At the end of the measurements, the animals were euthanized with an overdose of pentobarbital sodium (Euthasol 20 %) injected intracardially, followed by double-sided pneumothorax. Surgical procedures and technical specifications of the experimental setup for the in situ measurements have been described in detail elsewhere (Bernabei et al. 2015). The posterior crural compartment was exposed and biceps femoris and medial gastrocnemius muscles were removed. SO, LG and PL muscles were, as a group, dissected free from surrounding structures, preserving the bone insertions as well as the myofascial connections at the interface between the SO and $\hbox {LG}+\hbox {PL}$ muscle bellies (Fig. 1a). The neurovascular tracts supplying this muscle group were left intact. The distal tendon of SO was dissected free from the rest of the Achilles tendon. Kevlar wires were used to connect the proximal and distal tendons of $\hbox {LG}+\hbox {PL}$ as well as the distal tendon of SO to force transducers, which were positioned in such a way that forces could be measured in the muscle’s line of pull. It should be noted that the SO origin was left intact to preserve the original muscle position relative to the tibia; thus, SO proximal tendon force was not measured. A bipolar cuff electrode connected to a constant current source was folded around the sciatic nerve. The common peroneal nerve was severed, leaving only the tibial nerve and the sural branch intact.

#### Nerve stimulation

SO and $\hbox {LG}+\hbox {PL}$ were excited simultaneously via supramaximal stimulation of the sciatic nerve (0.4 ± 0.1 mA, 500 ms, 100 Hz). Two twitches were evoked 1.5 and 1 s before each tetanic stimulation. Muscles were allowed to recover for 2 min between subsequent stimulations.

#### Experimental protocol

The rat was mounted in the experimental setup by clamping the femur and the foot such that knee and ankle joints were kept at $90^{\circ }$, which was defined as the *reference position* $(P_{\mathrm{REF}})$. Markers were placed near the SO distal and $\hbox {LG}+\hbox {PL}$ proximal and distal tendons to apply different relative positions between SO and $\hbox {LG}+\hbox {PL}$ MTUs. To prevent history effects, multiple contractions at high and low lengths were elicited prior to data collection. Isometric forces exerted at all three tendons were measured simultaneously for different positions of $\hbox {LG}+\hbox {PL}$ relative to SO muscle $(P_{R})$. The proximal and distal $\hbox {LG}+\hbox {PL}$ tendons were repositioned in steps of 1 mm from $\hbox {P}_{\mathrm{REF}}-3\,\hbox {mm}$ in distal direction to $\hbox {P}_{\mathrm{REF}}+3\,\hbox {mm}$ in proximal direction (Fig 1b). $\hbox {LG}+\hbox {PL}$ tendons were repositioned together, while the distal and proximal tendons of SO were kept at the same position corresponding to $P_{\mathrm{REF}}$. Thus, MTU lengths were constant over all the imposed conditions, with SO and $\hbox {LG}+\hbox {PL}$ operating on the ascending region of their length–force curves (mean ± SD at $\hbox {P}_{\mathrm{REF}}: \hbox {F}_{\mathrm{LG+PL}}=11.5\pm 0.2\hbox {N}, \hbox {F}_{\mathrm{SO}}= 1.3\pm 0.5\hbox {N}$, Bernabei et al. 2015). Following the same experimental protocol, tendon forces were measured for three different conditions to estimate the individual contribution of inter and extramuscular pathways: (i) intact conditions (NO, $n=4$; TI, $n=7$); (ii) resection of proximal and distal intermuscular myofascial connections (NO, $n=4$; TI, $n=7$); (iii) complete isolation of synergists by resecting the neurovascular tract between SO and $\hbox {LG}+\hbox {PL}$ ($n=1$). It should be noted that the latter resection was possible only on rats with unaltered connectivity, as in the TI group the blood supply and nerve to SO could be no longer distinguished from the bundle of newly grown intermuscular connective tissue. Moreover, this procedure caused the SO to be no longer excitable. In a subset of animals, only distal myofascial connections were resected (NO, $n=3$) to assess the changes in tendon force when only the proximal, instead of proximal and distal, connective tissues pathway was involved (Fig. 1a).

**Fig. 2:**
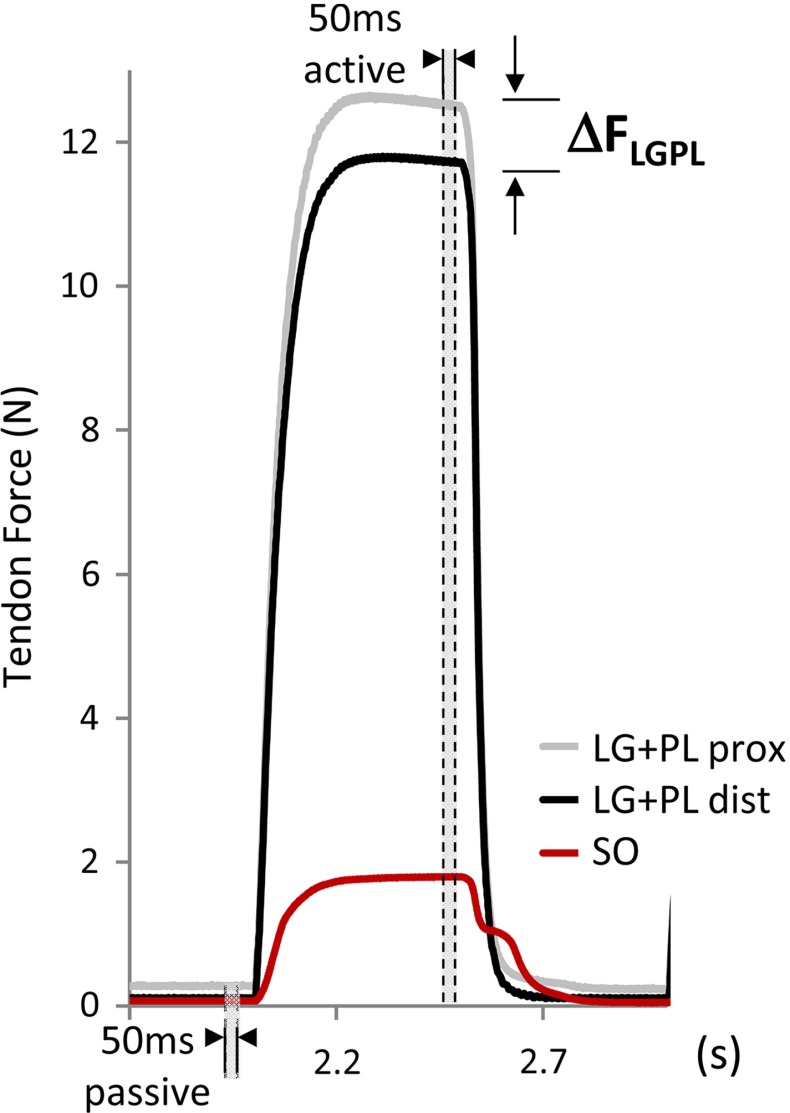
Exemplar waveforms of forces exerted at the tendons of SO and $\hbox {LG}+\hbox {PL}$. Passive and total forces at soleus distal (SO), $\hbox {LG}+\hbox {PL}$ proximal ($\hbox {LG}+\hbox {PL}$ prox) and $\hbox {LG}+\hbox {PL}$ distal ($\hbox {LG}+\hbox {PL}$ dist) tendons were measured simultaneously before (passive) and during (active) tetanic contraction. *Gray areas* indicate the 50 ms time-windows for extraction of forces during passive and active state of muscles. Mechanical interaction between SO and $\hbox {LG}+\hbox {PL}$ is noticeable in the first portion of SO relaxation, which is much quicker than what can be expected from the slow SO muscle fibers and, thus, attributed to relaxation of the faster $\hbox {LG}+\hbox {PL}$ muscle fibers

#### Analysis of experimental data

Isometric forces were assessed from the force-time series: passive force was assessed by calculating the mean over a 50 ms time window before the tetanic stimulation and total force was assessed by calculating the mean over the last 50 ms of the tetanic stimulation (Fig. 2). Two estimates of intermuscular mechanical interaction were assessed: with repositioning both proximal and distal $\hbox {LG}+\hbox {PL}$ tendons simultaneously, we calculated (i) changes in force exerted at the distal tendon of SO $(\Delta F_\mathrm{SO})$, with SO kept at a constant length and (ii) changes in the force difference between the proximal and distal tendons of $\hbox {LG}+\hbox {PL}$, with $\hbox {LG}+\hbox {PL}$ kept at a constant length. Changes in SO force were expressed relative to the value at $P_\mathrm{REF} (\Delta F_\mathrm{SO}=F_\mathrm{SO} (P_{R})-F_\mathrm{SO} (P_\mathrm{REF}))$. The $\hbox {LG}+\hbox {PL}$ force difference $(\Delta F_\mathrm{LGPL})$ was calculated by subtracting the force exerted distally from the force exerted proximally (i.e., $F_\mathrm{PROXIMAL}-F_\mathrm{DISTAL})$, so that a positive difference indicates a higher proximal force. This force difference is a direct measure of the magnitude of net epimuscular myofascial force transmission (Huijing and Baan 2001; Maas and Sandercock 2010). $\Delta F_\mathrm{LGPL}$ and $\Delta F_\mathrm{SO}$ as defined above will be referred to as the *calibration dataset*.

To test for effects of muscle relative position on proximal and distal $\hbox {LG}+\hbox {PL}$ forces, two-way analysis of variance (ANOVA) for repeated measures (within factor: muscle relative displacement) was performed. A linear regression analysis was used to evaluate the relation between $\Delta F_\mathrm{SO}$ and $\Delta F_\mathrm{LGPL}$ over $P_{R} \in [-3;+3]\,\hbox {mm}$. $\Delta F_\mathrm{SO}$ and $\Delta F_\mathrm{LGPL}$ were fitted with a polynomial function, with the order determined by stepwise regression. Generalized estimating equations (GEE) were used to test for significant differences between groups including the interactions with the factor groups (NO, TI), to test for differences in the slope of the models. *p* values <.05 were considered statistically significant.

### Modeling study

#### Inter- and extramuscular pathways of force transmission

The myofascial connective tissues at the interface between SO and $\hbox {LG}+\hbox {PL}$ muscle bellies, classified as the intermuscular pathway of force transmission (*INT*), were preserved and represented in the model as nonlinear elastic elements (Fig. 1c). This pathway was divided into a proximal and a distal part with respect to the neurovascular tract, which runs centrally between SO and LG muscle bellies (see Bernabei et al. 2016 for a detailed description). Bundles of connective tissues surrounding blood vessels and nerves reaching the posterior crural muscles can be divided in two main pathways for extramuscular force transmission (Fig. 1a). The first pathway (*NV1*) runs from the proximal-ventral side of LG to the dorsal side of SO. The second pathway (*NV2*) contains branches of the popliteal artery and tibial nerve running from the popliteal fossa to the proximal-dorsal surface of lateral and medial gastrocnemius muscle bellies. A clear depiction of the tibial nerve branches innervating the superficial muscles of the posterior crural compartment has been reported previously (Tijs et al. 2014). Although *NV1* was defined here as an extramuscular pathway of force transmission, because it ultimately leads forces outside the synergistic muscle group, it should be noted that it also contributes to intermuscular force transmission, since it establishes a direct connection between SO and LG epimysial layers.

#### Model description

A phenomenological lumped-parameter model was used to represent SO and $\hbox {LG}+\hbox {PL}$ interactions (Fig. 1c). Each muscle was described by a contractile element (CE) surrounded, both in series (SE) and in parallel (PE), by connective tissue structures. In particular, the mechanical properties of (i) intermuscular connective tissues *INT*, i.e., proximal and distal epimuscular myofascial connections and (ii) extramuscular connective tissue bundles *NV1* and *NV2*, were summarized by elastic elements with piecewise linear stiffness properties. A formal explanation of the equations and parameters used to describe the mechanical lumped-parameter system can be found in the “Appendix”. Briefly, the non-zero force balance at the tendons of a non-isolated muscle actuator can be seen as a direct measure of the amount of force transmitted inter- and extramuscularly to surrounding structures (Maas et al. 2004). Thus, $\Delta F_\mathrm{LGPL}$ was considered as an estimate of the force redirected from $\hbox {LG}+\hbox {PL}$ to neighboring structures via all the considered inter- and extramuscular connections. It should be noted that no direct information was available on the actual force vectors associated with the inter- and extramuscular pathways.

$F_\mathrm{proximal}$ and $F_\mathrm{distal}$ were measured and included in the model for the three different experimental conditions applied: (i) intact connective tissues pathways (*intact*), (ii) after resection of *INT* (*post I*) and (iii) after resection of *INT* and *NV1* (*post II*). With intact intermuscular and extramuscular connective tissue pathways, the balance of force produced by active muscles within the system shown in Fig. 1c is given by:

1 $$\begin{aligned}&\hbox {(node 1)} \qquad F_\mathrm{LGPL}^\mathrm{prox} -F_\mathrm{NV2}-F_\mathrm{LGPL}^\mathrm{PE}-F_\mathrm{LGPL}^\mathrm{CE} =0 \end{aligned}$$

2 $$\begin{aligned}&\hbox {(node 2)}\qquad F_\mathrm{LGPL}^\mathrm{dist}-F_\mathrm{LGPL}^\mathrm{PE}-F_\mathrm{LGPL}^\mathrm{CE} +F_\mathrm{INT} + F_\mathrm{NV1}=0 \end{aligned}$$

where *F* is the force redirected to the given node via the superscripted element. Because MTUs of $\hbox {LG}+\hbox {PL}$ and SO were kept at the same length between conditions and muscle contractions were isometric, $F_\mathrm{CE}$ and $F_\mathrm{PE}$ can be assumed to be constant and the forces in the system can be resolved with a static force equilibrium. Following progressive resection of connective tissues pathways, this system of equations was modified by setting the amount of force running through resected pathways to zero. It should be noted that proximal and distal *INT* pathways are represented in Eq. 2 by a single variable $F_\mathrm{INT}$. Solving Eqs. 1–2 for $F_\mathrm{INT}$ allowed to estimate the amount of force transmitted via intermuscular connective tissues pathways. A similar approach has been used previously to quantify myofascial force transmission between the multiple heads of the extensor digitorum longus muscle (Zhang and Gao 2014) and changes in the mechanical effect of flexor carpi ulnaris after dissection from surrounding connective tissues in the rat (Maas et al. 2013).

#### Stiffness estimates of intermuscular and extramuscular pathways

The stiffness estimate for each pathway of force transmission was based on input–output data obtained from the in situ experiments (*calibration dataset*). The mechanical properties of passive connective tissues, from tendon to blood vessels, can be characterized as quasi-static (Fung 1981), so that continuous force transmission changes occurring during dynamic tasks can be approximated by changes in isometric force measured at discrete relative positions. The behavior of collagen fibers, of different initial orientation, starting to stretch at different extensions can be represented with a piecewise linear function (Winters and Woo 1990). Therefore in this study, the estimate of stiffness $(\hat{K})$ for each inter- and extramuscular pathway of force transmission (*INT*, *NV1*, *NV2*) was defined as the ratio of force increase $(\Delta F)$ over a unit of relative displacement $(\Delta P_R)$.

Based on the system of equations which describes force equilibrium for different conditions of connective tissues resection, estimates of the force transmitted for each $\hbox {SO}/\hbox {LG}+\hbox {PL}$ relative displacement were calculated as:

3 $$\begin{aligned} \widehat{F_{\mathrm{INT}}}(P_{r})= & {} \Delta F_\mathrm{LGPL}^{\mathrm{intact}}-c*\Delta F_\mathrm{LGPL}^{\mathrm{post}\,\mathrm{I}} +\Delta F_\mathrm{LGPL}^{\mathrm{post}\,\mathrm{II}}*\left( {c-1} \right) \qquad \mathrm{[N]} \end{aligned}$$

4 $$\begin{aligned} \widehat{F_\mathrm{NV1}}(P_{r})= & {} \Delta F_\mathrm{LGPL}^{\mathrm{post}\,\mathrm{I}} -\Delta F_\mathrm{LGPL}^{\mathrm{post}\,\mathrm{II}} \qquad \hbox {[N]} \end{aligned}$$

5 $$\begin{aligned} \widehat{F_\mathrm{NV2}}(P_{r})= & {} \Delta F_\mathrm{LGPL}^{\mathrm{post}\,\mathrm{II}}\qquad \hbox {[N]} \end{aligned}$$

where $\Delta F_\mathrm{LGPL}$ is the force difference at $\hbox {LG}+\hbox {PL}$ proximal-distal tendons for the intact, *post I* and *post II* conditions. The dimensionless parameter *c* was set to 0.9 to describe the ratio between the length of *NV1* before and after resection of myofascial linkages (see “Appendix”). Sensitivity analysis with $c\in (0.05;1)$ resulted in a root-mean-square error corresponding to maximally 6.0 % of the average force transmitted via *INT* pathways over the imposed range of muscle displacements. To describe the distribution of force transmitted via non-myotendinous and myotendinous pathways, ratios of force transmitted via the intermuscular and extramuscular pathways and mean $\hbox {LG}+\hbox {PL}$ proximal tendon force were calculated $(\widehat{F_{\mathrm{INT}}}/F_\mathrm{LGPL}^\mathrm{prox}\,[\%]$ and $\widehat{F_\mathrm{NV1}}/F_\mathrm{LGPL}^\mathrm{prox}\, [\%], \widehat{F_\mathrm{NV2}}/F_\mathrm{LGPL}^\mathrm{prox}\, [\%]$).

#### Model validation

The lumped-parameter model was validated by comparing the predicted $\hbox {LG}+\hbox {PL}$ tendon force balance $(\Delta F_\mathrm{LGPL}^{\mathrm{mod}})$ with previously collected experimental data (Bernabei et al. 2016), here referred as the *testing dataset* $(\Delta F_\mathrm{LGPL}^\mathrm{exp})$. The experimental protocol used for measuring forces in the *testing dataset* differed from that used for the *calibration dataset*. For the *testing dataset*, $\hbox {LG}+\hbox {PL}$ force changes were measured applying a physiological range of lengths and relative displacements $(P_{R}^\mathrm{prox})$ between SO and $\hbox {LG}+\hbox {PL}$, with only $\hbox {LG}+\hbox {PL}$ proximal tendon repositioned from $P_{R}^\mathrm{prox}-3\,\hbox {mm}$ to $P_{R}^\mathrm{prox}+3\,\hbox {mm}$, while the distal tendons were kept at the same position. For the *calibration dataset* instead, both proximal and distal tendons of $\hbox {LG}+\hbox {PL}$ were repositioned together from $P_{R} - 3\,\hbox {mm}$ to $P_{R} +3\,\hbox {mm}$, with constant muscle lengths for $\hbox {LG}+\hbox {PL}$ and SO (see above). Predicted values of $\hbox {LG}+\hbox {PL}$ proximal-distal force difference were calculated as follows:

6 $$\begin{aligned} \Delta F_\mathrm{LGPL}^\mathrm{mod}(\Delta P_{R}^\mathrm{prox})= & {} \Delta F_\mathrm{LGPL}^\mathrm{mod}(P_\mathrm{REF})+r\cdot {\hat{K}}_\mathrm{INT}\Delta P_R^\mathrm{prox}\nonumber \\&+\,\hat{K}_\mathrm{NV1}\cdot \Delta P_{R}^\mathrm{prox}\nonumber \\&+\,\hat{K}_\mathrm{NV2}\cdot \Delta P_R^\mathrm{prox} \qquad \hbox {[N]} \end{aligned}$$

where $P_\mathrm{REF}$ corresponded to $P_{R}=0$; this $\hbox {SO}/\hbox {LG}+\hbox {PL}$ relative position represented the matching condition for muscle lengths and relative displacement between the *calibration dataset* and the *testing dataset*. $\Delta P_{R}^\mathrm{prox}$ defined the proximal displacement of $\hbox {LG}+\hbox {PL}$. The scale-factor *r* described the ratio of myofascially transmitted force when a proximal displacement only (*testing dataset*), rather than a proximal and distal displacement (*calibration dataset*), was applied.

#### Analysis of model results

ANOVA for repeated measurements (within factor: $\hbox {LG}+\hbox {PL}$ proximal tendon position, between factor: predicted vs. measured data) was used to test for differences between the values of $\hbox {LG}+\hbox {PL}$ force difference predicted by the model and those of the testing dataset. A Bland-Altman plot was used to evaluate the agreement between predicted and measured values of $\hbox {LG}+\hbox {PL}$ force differences. The bias was calculated as the mean difference between measured $(\Delta F_\mathrm{LGPL}^\mathrm{exp})$ and predicted force values $(\Delta F_\mathrm{LGPL}^\mathrm{mod})$.

## Results

### Regression analysis

#### Tetanic forces

**Fig. 3:**
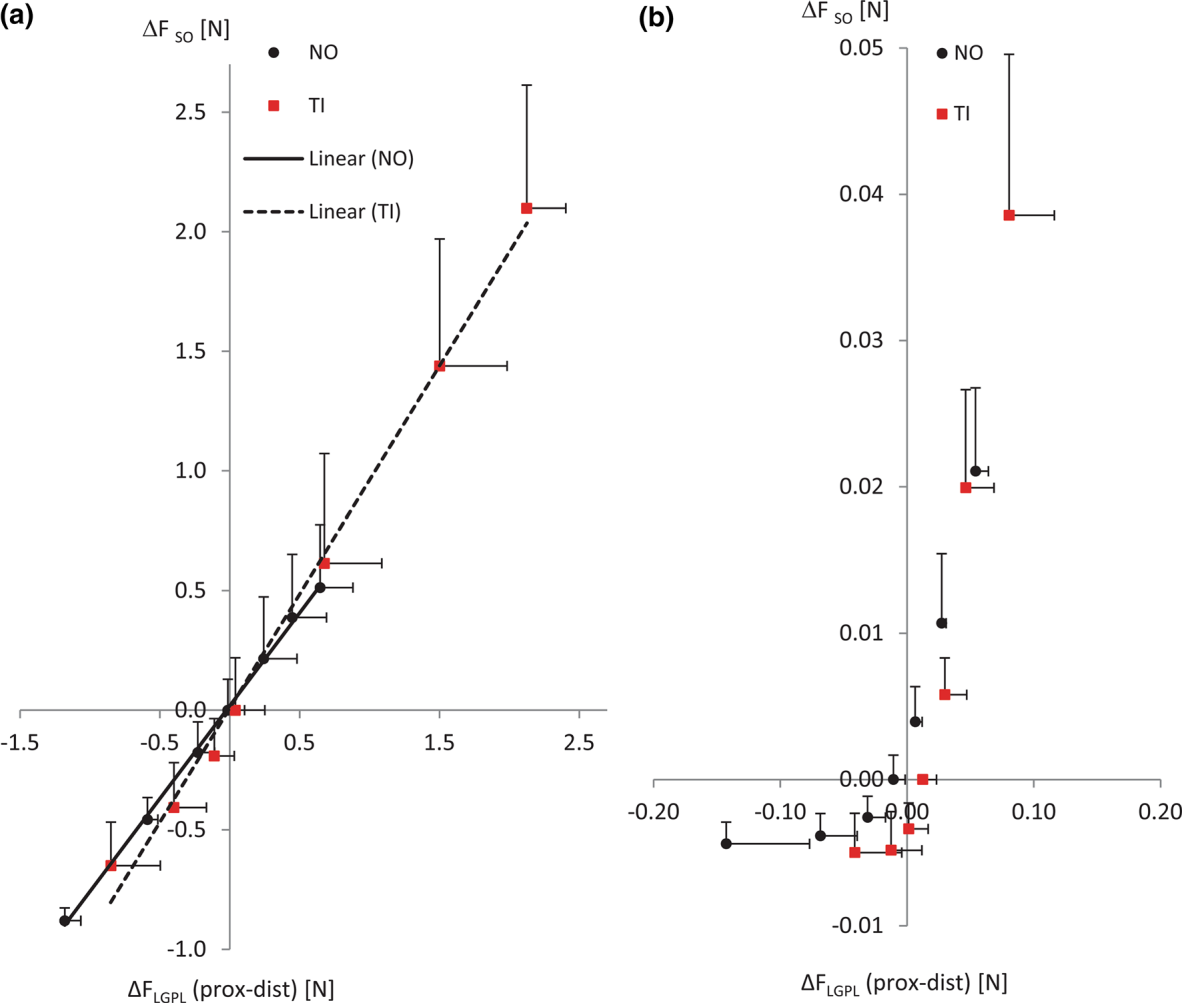
Comparison of SO and $\hbox {LG}+\hbox {PL}$ force discrepancies in active and passive state of muscles. Regression analysis between $\Delta F_{\mathrm{SO}}$ (mean $+$ SD) and $\Delta F_{\mathrm{LGPL}}$ (mean $+$ SD) with $\hbox {SO}/\hbox {LG}+\hbox {PL}$ relative displacement from $P_{R}=-3$ to $P_{R}=+3\,\hbox {mm}$. Total forces (**a**) and passive forces (**b**) are compared between the normal group (NO) and the tissue-integrating mesh group (TI). Linear regression lines are illustrated for each group (NO: *continuous line*; TI: *dashed line*). *R*-square values of the linear regression for the total force of NO and TI groups were 0.98 and 0.95, respectively. Passive forces in NO and TI were clearly nonlinear

A linear relationship between $\Delta F_\mathrm{LGPL}$ and $\Delta F_\mathrm{SO}$ was found (Fig. 3a). For both experimental groups, regression analysis revealed a positive slope (model fit: $p < .001$) with a high degree of correlation (NO: $R=0.98$; TI: $R=0.95$). The positive linear regression between SO and $\hbox {LG}+\hbox {PL}$ total forces suggests that the force discrepancy at $\hbox {LG}+\hbox {PL}$ tendons was balanced by the force exerted at SO distal tendon, which can be explained by force transmitted from $\hbox {LG}+\hbox {PL}$ to SO via connective tissues at their muscle belly interface.

GEE showed that the slope of the regression line for total force was significantly steeper in the TI group than in the NO group ($p=0.001$), suggesting that the ratio between the force transmitted via epimuscular pathways with respect to the myotendinous pathway was increased after the TI manipulation surgery. Compared to the NO group, the increased force range with the same applied relative displacement indicates that the stiffness of intermuscular linkages was enhanced in the TI group (NO: $\Delta F_\mathrm{LGPL}=0.51\hbox {N}$, TI: $\Delta F_\mathrm{LGPL}=2.12\hbox {N}$ at $P_{R}=+3\,\hbox {mm}$). Finally, it should be noted that $\Delta F_\mathrm{LGPL}$ reached 0 N at $P_{R}=0\hbox {mm}$ (REF position) and became negative for $P_{R}\in$ $[-3; 0]$ mm, which suggests a reversal of the sign of the epimuscular force transmission vector at $P_{R}=0\;\hbox {mm}$.

**Fig. 4:**
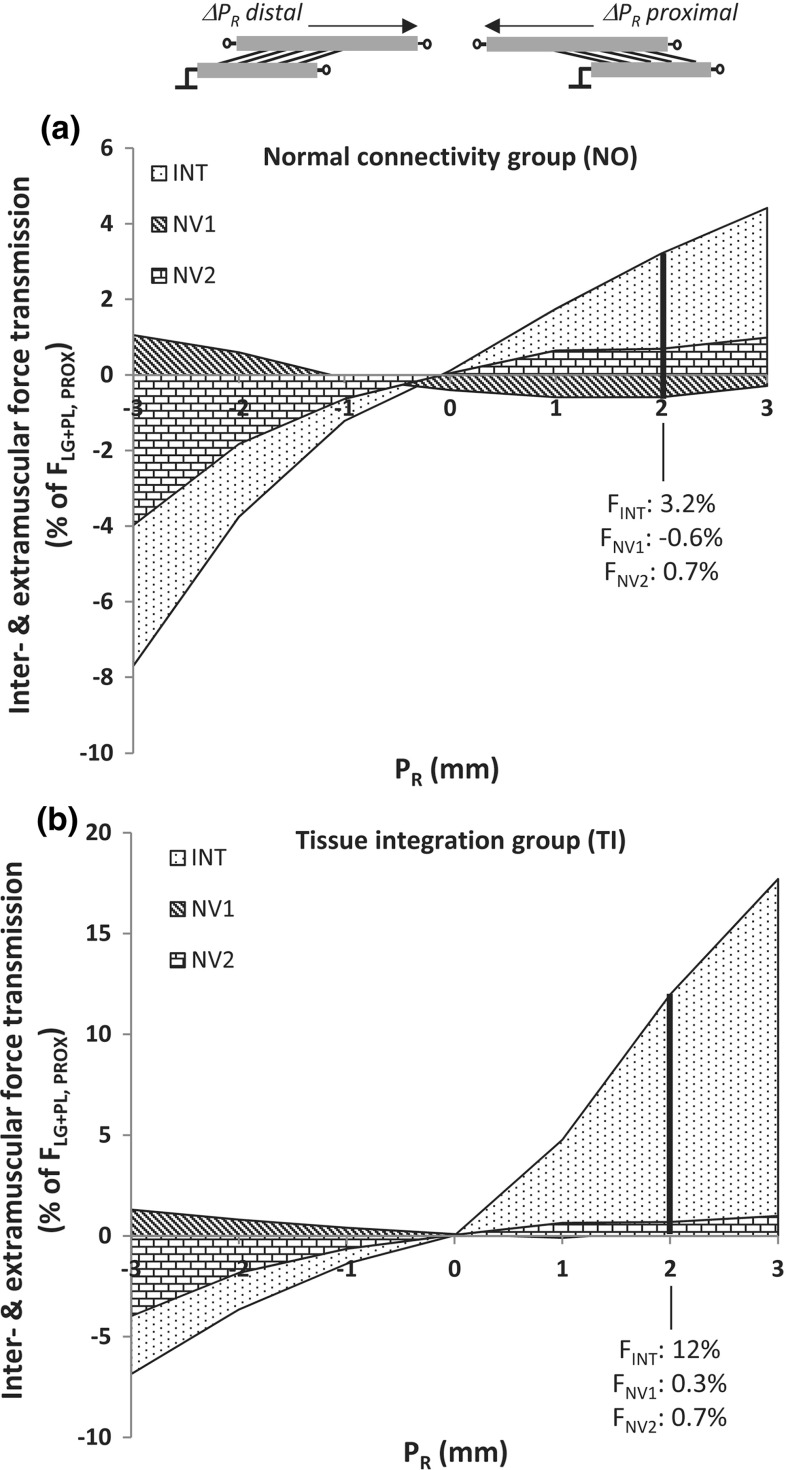
Epimuscular force transmission with normal and enhanced connective tissues. Distribution of epimuscularly transmitted force among intermuscular connective tissues (INT) and extramuscular neurovascular tracts (*NV*1, *NV*2), over each step of $\hbox {SO}/\hbox {LG}+\hbox {PL}$ MTUs displacement ($\Delta P_{R}$). Force values are expressed as a percentage relative to the $\hbox {LG}+\hbox {PL}$ proximal tendon total force ($\widehat{F}/F_{\mathrm{prox}}^{\mathrm{LG}}$ [%]) for the normal group (**a**) and the tissue-integration group (**b**). As an example, the distribution of force among the different pathways in respect to the myotendinous one, i.e., the proximal $\hbox {LG}+\hbox {PL}$ tendon, is shown for $P_{R}=2\,\hbox {mm}$

#### Passive forces

The relationship between passive $\Delta F_\mathrm{LGPL}$ and normalized SO force was clearly not linear for both NO and TI groups (Fig. 3b). This result suggests that in passive muscle conditions, the force difference at $\hbox {LG}+\hbox {PL}$ proximal and distal tendons was balanced only to some extent by force exerted at the SO distal tendon, especially for $P_{R}<0$ mm. Such non-exact force matching can be explained by force transmission via the proximal SO tendon and other non-muscular structures, which were not measured. As we considered the correspondence of $\Delta F_\mathrm{LGPL}$ with the normalized SO force an a priori condition for modeling force transmission via intermuscular connective tissues, estimates of epimuscular stiffness and tendon force predictions were not extended to the passive muscle state.

### Estimates of force transmission via intermuscular and extramuscular pathways

Force generated by $\hbox {LG}+\hbox {PL}$ muscle fibers was partially redirected to SO (via *INT* and *NV1*) and to non-muscular extramuscular structures (via NV2) with a variable extent as a function of relative position (Fig. 4). The amount of epimuscular myofascial force relative to myotendinous force $(\hat{F}/F_\mathrm{LGPL}^\mathrm{prox})$ for NO (Fig. 4a) and TI (Fig. 4b) was maximal at $P_{R}=-3\,\hbox {mm}$ (7.7 %) and at $P_{R}=+3\,\hbox {mm}$ (17.7 %), respectively. NV1 and NV2 contributed to transmit force up to 1.1 and −4.0 % of proximal $\hbox {F}_{\mathrm{LGPL}}$ at $P_{R}=-3\,\hbox {mm}$, respectively. It should be noted that the force transmission vector via *NV1* and *NV2* was oriented in opposite direction for most conditions. An overview of the stiffness estimates derived from the calibration dataset is reported in Table 1. These results show that the force distribution among SO and $\hbox {LG}+\hbox {PL}$ synergists is the result of a complex interaction between pathways of force transmission. The behavior of these pathways differed in terms of stiffness and configuration, i.e., angle and direction of the force transmission vector in response to changes in SO/$\hbox {LG}+\hbox {PL}$ relative position.

### Validation study

**Fig. 5:**
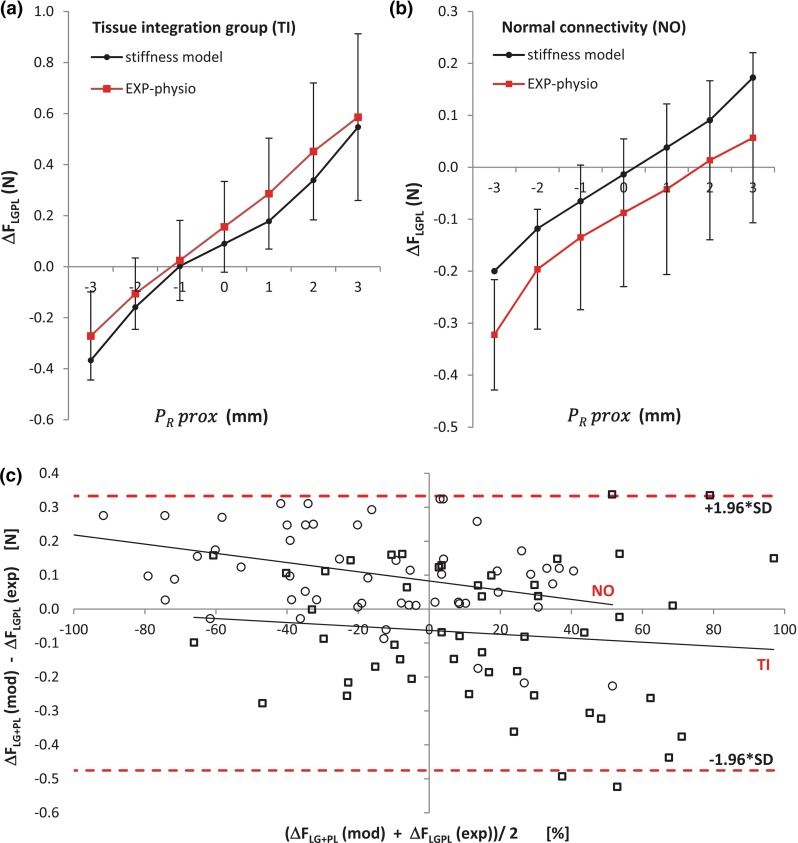
Model predictions versus measured experimental data. Validation of the epimuscular force transmission model ($n=12$, *calibration dataset*) using data from a previously reported in situ experiment as a *testing dataset* ($n=24$) in which physiological lengths and relative position of SO and $\hbox {LG}+\hbox {PL}$ were applied (Bernabei et al. 2016). Averaged predicted values (*black circles*) of $\hbox {LG}+\hbox {PL}$ proximal-distal force difference with repositioning $\hbox {LG}+\hbox {PL}$ proximal tendon $(\Delta P_R^{\mathrm{prox}})$ from $-3$ to $+3$ mm from $P_{\mathrm{REF}}$ are reported together with the corresponding experimental values (*red squares*, mean ± SD) for animals with enhanced connectivity (**a**, $n=7$) and animals with normal connectivity (**b**, $n=8$). Bland-Altman plot (**c**) showing the average bias (NO: 0.105N, TI: −0.071N) of the modeled data. Each point represents a force value estimated for all animals within NO (*circles*) and TI (*squares*) groups. Values of $\Delta F_{\mathrm{LG}+\mathrm{PL}}$ on the *x*-axis were expressed as a percentage of the total range of $\hbox {LG}+\hbox {PL}$ proximal-distal force difference $(\max (\Delta F_{\mathrm{LG}+\mathrm{PL}})-\min (\Delta F_{\mathrm{LG}+\mathrm{PL}}))$ to reduce variation between animals. The limits of agreement, expressed as the mean bias ± 1.96 SD, for individual force data are shown (lower limit: −0.475; upper limit: 0.366N)

ANOVA showed no significant differences between $\Delta F_\mathrm{LGPL}^\mathrm{exp}$ and $\Delta F_\mathrm{LGPL}^\mathrm{mod}$ ($p=.956$). In the *testing dataset*, $\hbox {LG}+\hbox {PL}$ proximal-distal force difference varied with $\hbox {LG}+\hbox {PL}$ proximal position $\Delta P_R^\mathrm{prox}$ for both NO and TI groups ($p<.001$, Fig. 5a, b, respectively); this force variation did not significantly differ between predicted and measured values ($p= .167$). The Bland-Altman plot shows a bias of the predicted dataset with respect to the measured one (NO: 0.106N, TI: −0.071N, or 13.1 and 8.8 % of the confidence interval, respectively). In particular, force values predicted by the model were overestimated at a low values of $\Delta F_\mathrm{LGPL}$ for NO and underestimated at high $\Delta F_\mathrm{LGPL}$ for TI (Fig. 5c). The limits of agreement between model and experimental data were comprised within a range of about 0.840 N (±1.96*SD $=$ 0.475; −0.366 N).

**Table 1:** Stiffness estimates (calibration dataset)

	Epimuscular pathway	${K}_{1}$ (mN/mm)	${K}_2$ (mN/mm)	${K}_{3}$ (mN/mm)
Control ($n=7$)	*INT*	239.8	171.7	143.9
	*NV1*	21.7	0.4	34.6
	*NV2*	56.6	30.8	17.1
Tissue-integration ($n=7$)	*INT*	549.7	842.5	681.1
	*NV1*	19.9	45.7	29.6
	*NV2*	69.4	5.6	35.5
${r}(\Delta {L}_{R})$: full vs. distal resection ratio ($n=3$)		0.07	0.13	0.21

$K_{N}$: parameters describing the stiffness of a given force pathway over a unit of relative displacement as defined in Eq. 7

## Discussion

We here presented a phenomenological multi-muscle model, which takes into account the intermuscular and extramuscular pathways of force transmission of the rat plantar-flexor muscles. Nonlinear estimates of stiffness for these pathways were derived based on in situ measurements of force transmission. We showed that such estimates can be used to predict the amount of force transmitted via non-myotendinous pathways at physiological lengths and relative positions of muscles. Although the magnitude of non-myotendinous force transmission effects in in vivo conditions are disputed (Herbert et al. 2008; Maas and Sandercock 2008; Tijs et al. 2015b), imaging studies in humans do imply relevant effects (Bojsen-Moller et al. 2010; Huijing et al. 2011; Tian et al. 2012; Yaman et al. 2013). Epimuscular connective tissues pathways may substantially affect muscle mechanics when these connections are either stiffer or more compliant than normal, e.g. because of surgery, injury or pathology (Yucesoy et al. 2007; Smeulders and Kreulen 2007; Huijing et al. 2010; Maas and Huijing 2012b).

**Fig. 6:**
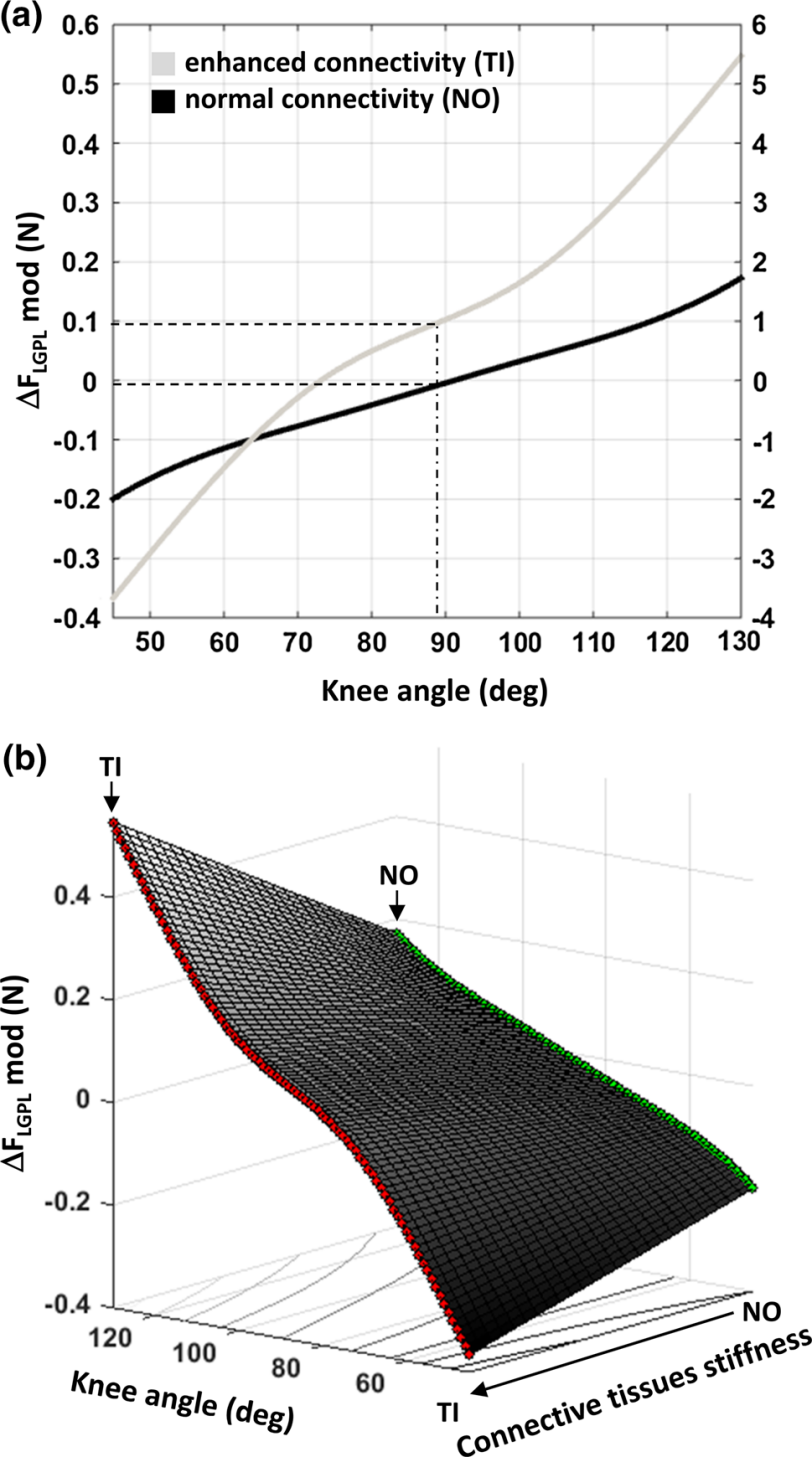
Epimuscular force transmission with knee angle extension. Force transmission estimates predicted by the model $(\Delta F_{\mathrm{LGPL}}^{\mathrm{mod}})$ plotted **a** as a function of knee angle corresponding to the applied proximal $\hbox {LG}+\hbox {PL}$ length changes $(P_R^{\mathrm{prox}})$ and **b** as a function of both knee angle and connective tissues stiffness. In the *top panel*, predicted values of intermuscular and extramuscular force transmission for a $90^{\circ }$ knee angle are highlighted for the evaluated intermuscular connectivity conditions, i.e., normal (NO) and enhanced connectivity (TI). The secondary axis expresses the predicted $\Delta F_{\mathrm{LGPL}}^{\mathrm{mod}}$ as a percentage of the mean $\hbox {LG}+\hbox {PL}$ proximal tendon force ($\hbox {F}_{\mathrm{LG}+\mathrm{PL}}$, proximal: $11.5\pm 0.2\hbox {N}$). In the bottom panel, linear interpolation of force transmission estimates between NO (*green*) and TI (*red*) over the range of corresponding knee angles

To our knowledge, this is the first model that relates the stiffness of connective tissues to changes in tendon forces, depending on the relative position of synergistic muscles. A difference between forces exerted simultaneously at the origin and insertion of a muscle is considered a direct measure of the force transmitted by a muscle to surrounding structures via non-myotendinous pathways (Huijing and Baan 2001; Maas et al. 2001). The relative position of a muscle with respect to surrounding muscles affects muscle function by altering the amount of force transmitted epimuscularly (Huijing and Baan 2003; Maas et al. 2004). In this study, we combined these features to obtain estimates of stiffness of intermuscular myofascial linkages and of neurovascular tracts. By deriving such estimates in a condition of normal (NO) and enhanced connectivity (TI), the extent of transmitted force could be related to the mechanical properties of these connective tissues. Moreover, given the correspondence between the imposed SO and $\hbox {LG}+\hbox {PL}$ relative positions with in vivo knee and ankle angles (Johnson et al. 2011), changes in force transmission can be represented as a function of joint angle (Fig. 6a). By linearly scaling the stiffness estimates between normal and enhanced connectivity conditions, the resulting three-dimensional function (Fig. 6b) allows a novel and concise representation of the epimuscular force transmission phenomena, which could be integrated in a more comprehensive musculoskeletal model. This would enable predictions of force changes at the tendons of non-isolated muscles in functional tasks when connective tissues are altered.

### Limitations

**Fig. 8:**
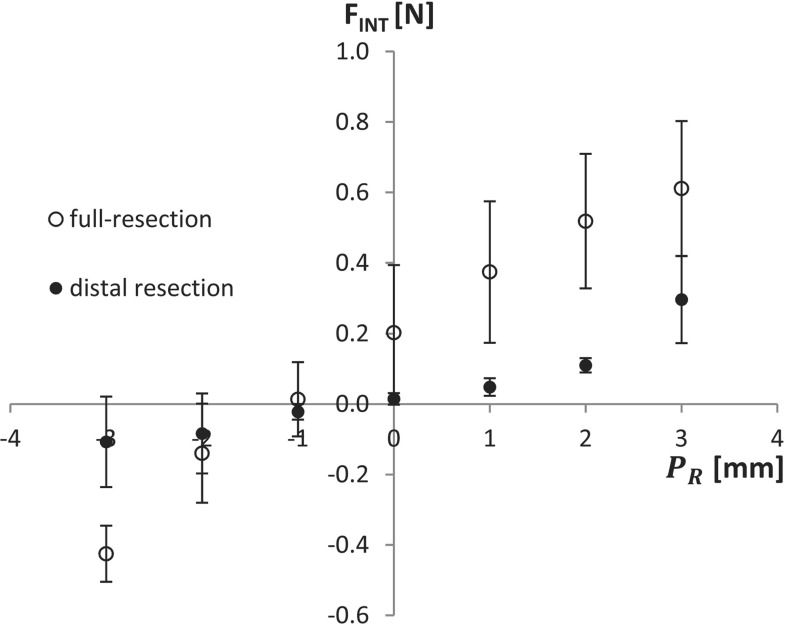
Effects of distal versus full resection on the estimate of intermuscular force transmission. Intermuscular force estimates (mean ± SD) over a range of muscle relative displacement obtained by comparing $\hbox {LG}+\hbox {PL}$ tendon forces before and after resection of all intermuscular connective tissues (*full resection*, *white circles*) or distal intermuscular connective tissues only (*distal resection*, *black circles*). The ratio between *full resection* and *distal resection* force values provided the *r* scaling factor at each considered $P_{R}$

Given the novelty of considering non-myotendinous pathways of force transmission in musculoskeletal modeling, a number of assumptions had to be made to reduce the complexity of the multi-muscle system and to isolate the effects of inter- and extramuscular connective tissues. First, unlike previous modeling studies (Maas et al. 2003; Yucesoy et al. 2007), we assumed the consequences of changes in the distribution of lengths of sarcomeres with changes of muscle relative position on muscle force to be negligible, an assumption that has been verified experimentally for the muscles considered here (Tijs et al. 2015a). Second, although local deformations of muscle bellies and differential strain of all force transmission pathways, from muscle fibers to tendons and epimuscular connective tissues, are expected (Finni et al. 2003; Blemker et al. 2005; Chi et al. 2010; Tijs et al. 2015b), we assumed that muscle relative position was the sole determinant of force changes in the muscle group. In fact, these local effects occur as a consequence of changes in the imposed relative positions, so that relative position was selected as a global descriptor of length changes for the whole mechanical chain. It is conceivable that changes of relative position occurring at both tendons simultaneously would elicit tension of non-myotendinous linkages to a higher extent and more homogeneously than considering proximal or distal positional changes only. In this regard, a comparison between the forces measured at $\hbox {LG}+\hbox {PL}$ tendon after resection of all intermuscular connective tissues and after resection of the distal intermuscular connective tissues only was included in the “Appendix” (Fig. 8). Third, the contribution of the Achilles tendon to the distribution of force within the muscle group (Sandercock 2000; Tijs et al. 2014) could not be taken into account, as the distal tendons of SO and lateral gastrocnemius were separated. This was necessary to measure individual tendon forces and to distinguish effects of non-myotendinous force pathways from the myotendinous ones. Finally, stiffness estimates were not representative of a passive muscle state, since this condition did not guarantee negligible deformation of muscle bellies during contraction. This was a necessary assumption of this model (see “Appendix”). In fact, it has been reported that changes in connective tissue layers between synergistic muscles are not equally expressed in passive and active states (Smeulders et al. 2002; Maas and Huijing 2012a; Bernabei et al. 2016). In agreement with these studies, we did not find equilibrium between $\Delta F_\mathrm{LGPL}$ and $\Delta F_\mathrm{SO}$ in the passive condition (Fig. 2b). Given the aforementioned assumptions and limitations, tendon forces predicted by this model should be carefully interpreted, especially when considering a different anatomy for connective tissues connections and various muscle activation levels.

### Implications for musculoskeletal modeling

When the assumption of mechanical independence of muscles cannot be verified, the output of a model based on this assumption may fail to predict forces in the musculoskeletal system represented. This has important implications for the experimental techniques used to model length–force characteristics of human muscles in vivo (Herzog and Keurs 1988; Hoang et al. 2007; Winter and Challis 2010). When using moment-angle data to reconstruct length–force curves of the muscles spanning a given joint, changes in relative position of a one-joint muscle respect to a two-joint muscle and the location of force measurement, i.e., proximal or distal tendons, can affect the result (Bernabei et al. 2015). The approach described in this study allows to explain inconsistent or variable outcomes of in vivo experiments when the assumption of mechanical independence is not guaranteed.

Considering in situ experiment instead, very often these are performed on non-isolated muscles or muscle compartments as well as dissected muscles still connected to their blood supply and innervation (Burke et al. 1976; Scott et al. 1996; Whitehead et al. 2001). These preparations are prone to effects of inter- and extramuscular force transmission, which may mask the actual mechanical behavior of the muscle fibers or introduce a force offset in between conditions with different relative positions between muscles. Given the results of the present study, a preliminary test on force differences at the tendons of a muscle with imposing different lengths or relative positions may help assessing the extent of myofascial effects. Alternatively, we showed that passive elastic elements are sufficient to model muscles’ interaction when the assumption of mechanical independence cannot be verified. Such a conclusion is in agreement with the study of Devasahayam and Sandercock (1992), where extramuscular passive elements were introduced in a simple Hill-type model with isolated actuators to explain force discrepancies at both ends of a muscle.

FE multi-muscle models have been used to represent effects of extramuscular force transmission on muscle mechanics with changes of muscles relative position (Maas et al. 2003; Yucesoy et al. 2003). However, the FE method is unnecessarily complex for describing global tendon forces, and too computationally demanding to be embedded in large-scale biomechanical models of the musculoskeletal system. In contrast, the model described here can be used to predict force changes at the muscle and muscle group level, thus matching the output of musculoskeletal models representing muscle function at a macroscopic level.

### Implications for modeling of in vivo human function and pathology

In this study, stiffness estimates were calculated for normal connective tissues and for a condition in which intermuscular connectivity was enhanced. Such a condition simulates the effects of altered connective tissues as a consequence of trauma, invasive surgical interventions, muscle-tendon injury, aging and pathologies such as muscle spasticity affecting connective tissues. For instance, scar tissue formation following rupture of muscle fibers and of the surrounding connective tissues framework has been associated with shearing type of muscle injuries (Kääriäinen et al. 2000). Scar tissue may result also in a reduction of the overall muscle belly displacement and a localized increase of tissue strain (Silder et al. 2010). We have shown that such enhancement of intermuscular connections alters the mechanical interaction between synergistic muscles (Bernabei et al. 2016). Similar mechanical effects of scar tissue may be expected following orthopedic surgical interventions involving disruption of muscle fascia or other connective tissues (e.g. fasciotomy, aponeurotomy and tenotomy). In fact, several studies have reported changes of intermuscular interaction following resection of connective tissues that served as pathways of force transmission (Smeulders et al. 2002; Maas et al. 2013; Ateş et al. 2013). A first attempt to model effects of altered non-myotendinous force transmission due to scar tissue development after tendon-transfer surgery was reported by Andersen (2009). In his extensive musculoskeletal model, a constant factor describes how muscle force is split between the transferred rectus femoris muscle (RF) and the vasti muscles via scar tissue. However, fibrotic connective tissues were defined as spurious muscle elements capable of producing a percentage of the normal muscle strength, regardless of the relative position between RF and patella. In this respect, estimates of inter- and extramuscular stiffness could complement Andersen’s model by taking into account a more realistic nonlinear stiffness factor, dependent on muscles’ relative position and scaled to the extent of tissue-integration post-intervention (see Fig. 5b). As opposite to the increased force transmission with stiffer epimuscular tissues, a degradation of mechanical properties of connective tissues has been proposed to explain reduced muscle force in Ehlers-Danlos patients (Huijing et al. 2010). Effects of degraded mechanical properties of connective tissues, e.g. a lower stiffness, could be extrapolated from the model presented here, which would provide a qualitative description of the tendon force changes as a consequence of impaired force transmission.

The complex organization of the connective tissues network surrounding muscles requires a detailed description of the anatomy of force pathways. Recently, subject-specific models to predict effects of orthopedic surgery have been proposed to overcome variability among patients in terms of anatomy, activity levels, loading conditions etc. (Carbone et al. 2015). In this regard, the approach described here would help obtaining more accurate estimates of human function when connective tissues pathways are affected or impaired after muscle-tendon injury or orthopedic surgery. Further development is required to scale the non-myotendinous stiffness estimates to human muscles, also taking into account the organization and heterogeneity of inter- and extramuscular linkages between human muscle groups. In addition, the magnitude of non-myotendinous force transmission appears to be related to the extent of muscle activation and co-activation as suggested by differences between passive and active states (Finni et al. 2015; Tijs 2015). Therefore, scaling of force transmission estimates with muscle activation levels may be needed to integrate this approach into a musculoskeletal model driven by neuromuscular activation time series.

## Conclusions

The multi-muscle phenomenological model presented here provides a tool for studying effects of epimuscular force transmission on synergistic muscles force production. It improves our representation of muscle mechanics by implementing the connective tissues network, which surrounds muscles and can transmit the force produced within the muscle to other muscles and to non-muscular structures. Thus, this approach enables predictions of tendon forces in musculoskeletal models when the common assumption of muscle mechanical independence is challenged. Direct applications involve simulation of scar tissue development and prediction of surgical outcomes, where the effects of altered epimuscular myofascial force transmission may be substantial.

## Figures and Tables

**Fig. 7:**
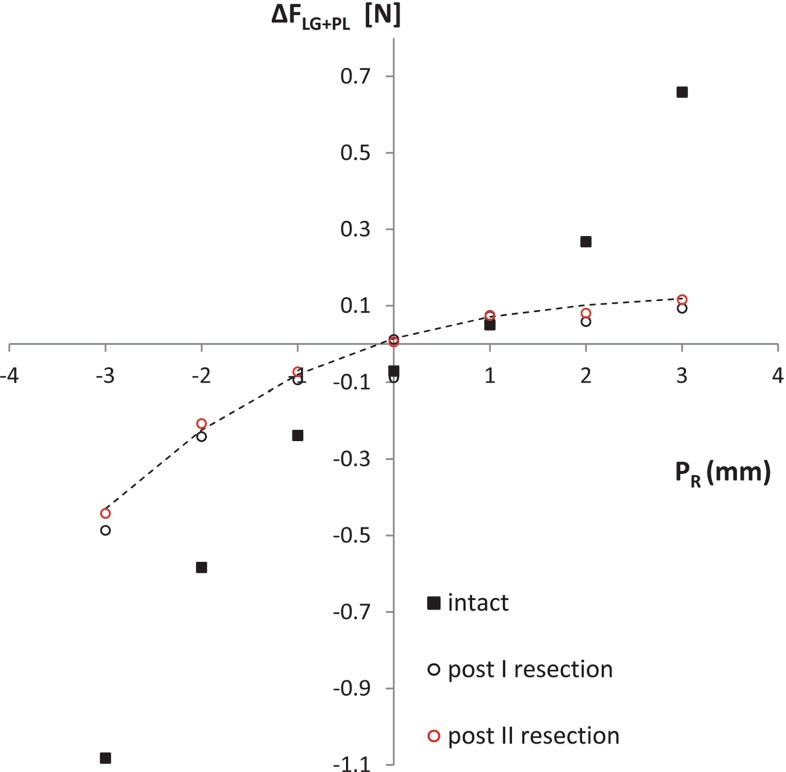
$\hbox {LG}+\hbox {PL}$ proximal-distal force difference $(\Delta {F}_{\mathrm{LGPL}})$ with progressive resection of connective tissues pathways. $\Delta F_{\mathrm{LGPL}}$ with intact connective tissues (*intact*), after resection of intermuscular connective tissues (*post I*) and after resection of all intermuscular and extramuscular force pathways (*post II*), but the neurovascular tract running proximally from $\hbox {LG}\,+\,\hbox {PL}$ (*NV2*). Residual $\hbox {LG}\,+\,\hbox {PL}$ proximal-distal force difference data points over the applied range of $\hbox {SO}/\hbox {LG}+\hbox {PL}$ relative displacement after resection of intermuscular pathways and the neurovascular tract running between SO and $\hbox {LG}+\hbox {PL}$ (*post II* resection) were interpolated with a III-order polynomial (*dashed line*, $n=1$)

## Appendix

The stiffness estimate assessed in this study is a lumped representation of the force redirected from one muscle to the tendon of a neighboring muscle via intermuscular connective tissues (*INT*), blood vessels and nerves (*NV1*, *NV2*). The constituent equation for the intermuscular and extramuscular connectivity elements was defined as:

7 $$\begin{aligned} \hat{K}=\frac{\Delta F}{\Delta P_R}=\left\{ {{\begin{array}{l@{\quad }l} K_1 &{}\,0<\Delta P_R \le 1\,\hbox {mm} \\ K_{2} &{}\,1<\Delta P_R \le 2\,\hbox {mm} \\ K_{3} &{}\,2<\Delta P_R \le 3\,\hbox {mm} \\ \end{array}}} \right. \quad \hbox {[N/mm]} \end{aligned}$$

where $\Delta F$ is force change, $\Delta P_{R}$ is the change in relative position between connected muscles and $\hat{K}$ is the parameter describing the stiffness of a given force pathway. Connective tissues, ranging from tendon to skin to blood vessels, tend to have quasi-static mechanical properties: the stiffness increases fairly linearly with force over the primary operating range (Fung 1981; Winters and Woo 1990). Thus, the lumped stiffness of intermuscular connections was modeled as dependent on the synergists’ relative position $P_{R}$ for which a piecewise linear relation was chosen (Eq. 7). Each muscle was divided into a proximal and a distal node to conveniently represent myofascial connective tissue anchoring points (Fig. 1c).

### Model assumptions

- Muscle bellies were assumed to undergo negligible deformation during contraction.
- Sarcomere length changes were assumed to be negligible as muscles were kept at constant MTU length, so that constant isometric force was obtained at each $P_{R}$. It should be noted that sarcomere length distribution may vary, at least in passive conditions, for different imposed relative positions between muscles (Tijs et al. 2015b).
- Length changes of underlying structures (*INT*, *NV1*, *NV2 *pathways) were considered proportional to muscles’ relative displacement:
  8 $$\begin{aligned} \Delta l\propto \Delta P_{R} \end{aligned}$$
- Mechanical properties of NV2 extramuscular connective tissues are preserved after resections:
  9 $$\begin{aligned} \hat{K}_\mathrm{NV2}^{\mathrm{post}\,\mathrm{II}} =\hat{K} _\mathrm{NV2}^{\mathrm{post}\,\mathrm{I}} =\hat{K}_\mathrm{NV2}^\mathrm{intact} \end{aligned}$$
- Myofascial resection at SO/LG+PL interface (*INT*/*NV1 *pathways) does not change the length of the extramuscular tissues pathway outside SO/LG+PL interface (*NV2*):
  10 $$\begin{aligned} \Delta l_\mathrm{NV2}^\mathrm{intact} =\Delta l_\mathrm{NV2}^{\mathrm{post}\,\mathrm{I}} =\Delta l_\mathrm{NV2}^{\mathrm{post}\,\mathrm{II}} \end{aligned}$$
- The ratio between length changes of *NV1* before (*intact*) and after resection of the myofascial linkages (*post I*) was assumed to be a constant:
  11 $$\begin{aligned} \frac{\Delta \hbox {l}_{\mathrm{NV1}}^{\mathrm{intact}}}{\Delta \hbox {l}_{\mathrm{NV1}} ^{{\mathrm{post}}\,{\mathrm{I}}}}=\hbox {c} \end{aligned}$$

Sensitivity analysis on *c* was performed by calculating the residuals between the range of predicted force transmission values $\widehat{F_\mathrm{INT}}$, as defined in Eq. 21, for $c\in (0.05;1.00)$ and the $\widehat{F_\mathrm{INT}}$ obtained by setting $c=0.9$, corresponding to minimal changes of $l_\mathrm{NV1}$ after resection of *INT* (*post I*). Analysis of root-mean-square of residuals revealed that the difference obtained by assuming $c=0.9$ was up to 6.0 % of the average $\widehat{F_\mathrm{INT}}$ over the imposed range $\Delta P_{R}=6\hbox {mm}$.

### Stiffness estimates assessment

With intact intermuscular and extramuscular connective tissue pathways, the balance of forces produced by active muscles within the system shown in Fig. 1 can be written as:

12 $$\begin{aligned}&\hbox {(node 1)}\qquad \! F_\mathrm{LGPL}^\mathrm{prox} -F_\mathrm{NV2}-F_\mathrm{LGPL}^\mathrm{PE} -F_\mathrm{LGPL}^\mathrm{CE} =0 \end{aligned}$$

13 $$\begin{aligned}&\hbox {(node 2)} \qquad \! F_\mathrm{LGPL}^\mathrm{dist} -F_\mathrm{LGPL}^\mathrm{PE} -F_\mathrm{LGPL}^\mathrm{CE} +F_\mathrm{INT}+F_\mathrm{NV1}=0 \end{aligned}$$

The aforementioned conditions assessed in the in situ experiment were: (i) intact connective tissues (*intact*), (ii) full resection of intermuscular myofascial connective tissues (*INT* resection, *post I*) and (iii) removal of all connections between SO and $\hbox {LG}+\hbox {PL}$ (*INT* and *NV1 *resection, *post II*). Given the isometric conditions, accelerations were inherently equal to zero $(\dot{\hbox {u}}_{\mathrm{prox}}^{LGPL}=\dot{\hbox {u}}_{\mathrm{prox}}^{\mathrm{LGPL}})$, therefore:

14 $$\begin{aligned} \Delta F_\mathrm{LGPL}^\mathrm{intact} =F_\mathrm{INT}^\mathrm{intact} +F_\mathrm{NV1}^\mathrm{intact} +F_\mathrm{NV2}^\mathrm{intact} \end{aligned}$$

After resection of the intermuscular connective tissues (the *INT* pathway), this becomes

15 $$\begin{aligned} (F_\mathrm{INT} \rightarrow 0) \quad \Delta F_\mathrm{LGPL}^{\mathrm{post}\,\mathrm{I}} =F_\mathrm{NV1}^{\mathrm{post}\,\mathrm{I}} +F_\mathrm{NV2}^{\mathrm{post}\,\mathrm{I}} \end{aligned}$$

After resection of both intermuscular and extramuscular pathways of force transmission (*INT+NV1*, *post II* condition, Fig. 7) and considering Eq. 11, the force equilibrium can be further reduced to

16 $$\begin{aligned} (F_\mathrm{INT} \rightarrow 0, F_\mathrm{NV1} \rightarrow 0) \quad \Delta F_\mathrm{LGPL}^\mathrm{postII} =F_\mathrm{NV2}^\mathrm{postII} \end{aligned}$$

The residual $\hbox {LG}+\hbox {PL}$ proximal-distal force difference $(F_\mathrm{NV2}^{postII})$ with $P_{R}\in$ $(-3;+3)$ mm was measured and interpolated with a III-order polynomial (Fig. 7, dotted line). Considering Eqs. 9, 15 and Eq. 16 becomes:

17 $$\begin{aligned} \Delta F_\mathrm{LGPL}^{\mathrm{post}\,\mathrm{I}}=F_\mathrm{NV1}^{\mathrm{post}\,\mathrm{I}} +\frac{\Delta F_\mathrm{LGPL}^\mathrm{postII}}{\Delta l_\mathrm{NV2}^\mathrm{postII}}\cdot \Delta l_\mathrm{NV2}^{\mathrm{post}\,\mathrm{I}} \end{aligned}$$

Simplifying this solution by considering Eq. 10:

18 $$\begin{aligned} F_\mathrm{NV1}^{\mathrm{post}\,\mathrm{I}} =\Delta F_\mathrm{LGPL}^{\mathrm{post}\,\mathrm{I}}-\Delta F_\mathrm{LGPL}^{\mathrm{post}\,\mathrm{II}} \end{aligned}$$

By adding this terms to the force equilibrium before resection (*intact* condition), Eq. 14 becomes:

19 $$\begin{aligned} \Delta F_\mathrm{LGPL}^\mathrm{intact}= & {} F_\mathrm{INT}^\mathrm{intact} +\frac{\Delta F_\mathrm{LGPL}^{\mathrm{post}\,\mathrm{I}} -\Delta F_\mathrm{LGPL}^{\mathrm{post}\,\mathrm{II}} }{\Delta l_\mathrm{NV1}^{\mathrm{post}\,\mathrm{I}} }\cdot \Delta l_\mathrm{NV1}^{\mathrm{post}\,\mathrm{I}}+\Delta F_\mathrm{LGPL}^{\mathrm{post}\,\mathrm{II}} \end{aligned}$$

which considering Eq. 11, can be written as:

20 $$\begin{aligned} F_\mathrm{INT}^\mathrm{intact} =\Delta F_\mathrm{LGPL}^\mathrm{intact} -c\cdot \Delta F_\mathrm{LGPL}^{\mathrm{post}\,\mathrm{I}} +\Delta F_\mathrm{LGPL}^{\mathrm{post}\,\mathrm{II}} \cdot (c-1) \end{aligned}$$

The force transmitted via intermuscular connective tissue pathways (*INT*) between SO and $\hbox {LG}+\hbox {PL}$ as a function of synergists’ relative position $(P_{R})$ can now be written as

21 $$\begin{aligned} \widehat{F_\mathrm{INT}}(P_{r})=\Delta F_\mathrm{LGPL}^\mathrm{intact} -c*\Delta F_\mathrm{LGPL}^{\mathrm{post}\,\mathrm{I}}+\Delta F_\mathrm{LGPL}^{\mathrm{post}\,\mathrm{II}} *(c-1) \end{aligned}$$

Thus, according to Eq. 7, the estimate of stiffness associated with intermuscular myofascial linkages was defined as

22 $$\begin{aligned} \hat{K}_\mathrm{INT} (P_{r})=\frac{\widehat{F_\mathrm{INT}}(P_{r}) -\widehat{F_\mathrm{INT}}(P_{r}-1)}{\Delta P_{r}} \end{aligned}$$

While the force transmitted via the intermuscular neurovascular tract between SO and $\hbox {LG}+\hbox {PL}$ (*NV1*) as a function of synergists’ relative position change $(P_{r})$ can be written as

$$\begin{aligned} \widehat{F_\mathrm{NV1}}(P_{r})=\Delta F_\mathrm{LGPL}^{\mathrm{post}\,\mathrm{I}}-\Delta F_\mathrm{LGPL}^{\mathrm{post}\,\mathrm{II}} \end{aligned}$$

Thus, the relative estimate of stiffness was defined as

23 $$\begin{aligned} \hat{K}_\mathrm{NV1}(P_{r})=\frac{\widehat{F_\mathrm{NV1}} (P_{r})-\widehat{F_\mathrm{NV1}}(P_{r}-1)}{\Delta P_{r}} \end{aligned}$$

### Model validation

The validation of the model obtained by the *calibration dataset* was performed by comparing predictions of $\Delta F_\mathrm{LGPL}$ ($\Delta F_\mathrm{LGPL}^\mathrm{mod}$) with experimentally measured values of $\Delta F_\mathrm{LGPL}$ ($\Delta F_\mathrm{LGPL}^\mathrm{exp}$), which belong to a *testing* *dataset*. Such dataset was obtained from the same animals, but applying a different protocol that spans physiological muscle lengths and relative positions changes. The predicted $\hbox {LG}+\hbox {PL}$ force difference which describes the amount of force redirected inter- and extramuscularly was defined as:

24 $$\begin{aligned} \Delta F_\mathrm{LGPL} (\Delta P_{R})=\Delta F_\mathrm{LGPL}(P_\mathrm{REF}) +F_\mathrm{INT}+F_\mathrm{NV1}+F_\mathrm{NV2} \end{aligned}$$

We expected that, for physiological muscle lengths and relative position of SO/LG+PL, a more limited involvement of intermuscular and extramuscular pathways would result in a reduced magnitude of force transmission (Bernabei et al. 2015). This was taken into account by scaling the stiffness descriptor of *INT* pathway with a function of the proximal and distal relative displacement between muscles: $r(\Delta L_{R})$. This function was determined experimentally by resecting only the distal portion of the intermuscular connective tissues and calculating the ratio of changes in force between full resection and distal resection for each relative position (Fig. 8, $n=3$). The implicit assumption is that the absence of distal connections would resemble the condition in which only the proximal connections are involved in the force transmission pathway. Therefore, with a proximal lengthening of $\hbox {LG}+\hbox {PL}$ muscle that corresponds to physiological conditions, the predicted $\hbox {LG}+\hbox {PL}$ force difference was calculated as:

$$\begin{aligned} \Delta F_\mathrm{LGPL}^\mathrm{mod}(\Delta P_{R}^\mathrm{prox})= & {} \Delta F_\mathrm{LGPL}^\mathrm{mod}(P_\mathrm{REF})+r\cdot \hat{K}_\mathrm{INT} \cdot \Delta P_{R}^\mathrm{prox}\\&+\,\hat{K}_\mathrm{NV1}\cdot \Delta P_R^\mathrm{prox}+\hat{K}_\mathrm{NV2} \cdot \Delta P_{R}^\mathrm{prox} \end{aligned}$$

## References

[CR1] Almeida-Silveira MI, Lambertz D, Pérot C, Goubel F (2000). Changes in stiffness induced by hindlimb suspension in rat Achilles tendon. Eur J Appl Physiol.

[CR2] Andersen MS (2009). Kinematically over-determinate musculoskeletal systems.

[CR3] Ateş F, Özdeşlik RN, Huijing P, Yucesoy C (2013). Muscle lengthening surgery causes differential acute mechanical effects in both targeted and non-targeted synergistic muscles. J Electromyogr Kinesiol.

[CR4] Bernabei M, van Dieën JH, Baan GC, Maas H (2015). Significant mechanical interactions at physiological lengths and relative positions of rat plantar flexors. J Appl Physiol.

[CR5] Bernabei M, van Dieën JH, Maas H (2016). Altered mechanical interaction between rat plantar flexors due to changes in intermuscular connectivity. Scand J Med Sci Sports.

[CR6] Blemker SS, Pinsky PM, Delp SL (2005). A 3D model of muscle reveals the causes of nonuniform strains in the biceps brachii. J Biomech.

[CR7] Bojsen-Moller J, Schwartz S, Kalliokoski KK (2010). Intermuscular force transmission between human plantarflexor muscles in vivo. J Appl Physiol.

[CR8] Booth C (2001). Collagen accumulation in muscles of children with cerebral palsy and correlation with severity of spasticity. Dev Med Child Neurol.

[CR9] Burke RE, Rudomin P, Zajac FE (1976). The effect of activation history on tension production by individual muscle units. Brain Res.

[CR10] Carbone V, Fluit R, Pellikaan P (2015). TLEM 2.0—a comprehensive musculoskeletal geometry dataset for subject-specific modeling of lower extremity. J Biomech.

[CR11] Chi SW, Hodgson J, Chen JS (2010). Finite element modeling reveals complex strain mechanics in the aponeuroses of contracting skeletal muscle. J Biomech.

[CR12] Close R (1964). Dynamic properties of fast and slow skeletal muscles of the rat during development. J Physiol.

[CR13] Correa TA, Baker R, Graham HK, Pandy MG (2011). Accuracy of generic musculoskeletal models in predicting the functional roles of muscles in human gait. J Biomech.

[CR14] Devasahayam SR, Sandercock TG (1992). Velocity of shortening of single motor units from rat soleus. J Neurophysiol.

[CR15] Ettema GJ, Huijing PA, van Ingen Schenau GJ, de Haan A (1990). Effects of prestretch at the onset of stimulation on mechanical work output of rat medial gastrocnemius muscle-tendon complex. J Exp Biol.

[CR16] Finni T, Cronin NJ, Mayfield D (2015). Effects of muscle activation on shear between human soleus and gastrocnemius muscles. Scand J Med Sci.

[CR17] Finni T, Hodgson JA, Lai AM (2003). Nonuniform strain of human soleus aponeurosis-tendon complex during submaximal voluntary contractions in vivo. J Appl Physiol.

[CR18] Fung YC (1981). Biomechanics.

[CR19] Garrett WEJ, Nikolaou PK, Ribbeck BM (1988). The effect of muscle architecture on the biomechanical failure properties of skeletal muscle under passive extension. Am J Sports Med.

[CR20] Herbert RD, Hoang PD, Gandevia SC (2008). Are muscles mechanically independent?. J Appl Physiol.

[CR21] Herzog W, ter Keurs HE (1988). A method for the determination of the force-length relation of selected in-vivo human skeletal muscles. Pflugers Arch.

[CR22] Higham TE, Biewener AA (2011). Functional and architectural complexity within and between muscles: regional variation and intermuscular force transmission. Philos Trans R Soc Lond B Biol Sci.

[CR23] Hoang PD, Herbert RD, Todd G (2007). Passive mechanical properties of human gastrocnemius muscle tendon units, muscle fascicles and tendons in vivo. J Exp Biol.

[CR24] Huijing P, Baan GC (2003). Myofascial force transmission: muscle relative position and length determine agonist and synergist muscle force. J Appl Physiol.

[CR25] Huijing P, Baan GC, Rebel GT (1998). Non-myotendinous force transmission in rat extensor digitorum longus muscle. J Exp Biol.

[CR26] Huijing PA (2009). Epimuscular myofascial force transmission: a historical review and implications for new research. International Society of Biomechanics Muybridge Award Lecture, Taipei, 2007. J Biomech.

[CR27] Huijing PA, Baan GC (2001). Extramuscular myofascial force transmission within the rat anterior tibial compartment: proximo-distal differences in muscle force. Acta Physiol Scand.

[CR28] Huijing PA, Voermans NC, Baan GC (2010). Muscle characteristics and altered myofascial force transmission in tenascin-X-deficient mice, a mouse model of Ehlers-Danlos syndrome. J Appl Physiol.

[CR29] Huijing PA, Yaman A, Ozturk C, Yucesoy CA (2011). Effects of knee joint angle on global and local strains within human triceps surae muscle: MRI analysis indicating in vivo myofascial force transmission between synergistic muscles. Surg Radiol Anat.

[CR30] Jacobs R, Bobbert MF, van Ingen Schenau GJ (1996). Mechanical output from individual muscles during explosive leg extensions: the role of biarticular muscles. J Biomech.

[CR31] Johnson WL, Jindrich DL, Zhong H (2011). Application of a rat hindlimb model: a prediction of force spaces reachable through stimulation of nerve fascicles. IEEE Trans Biomed Eng.

[CR32] Kääriäinen M, Järvinen T, Järvinen M (2000). Relation between myofibers and connective tissue during muscle injury repair. Scand J Med Sci Sports.

[CR33] Kovanen V, Suominen H, Peltonen L (1987) Effects of aging and life-long physical training on collagen in slow and fast skeletal muscle in rats. Cell Tissue Res 248(2):247–255 10.1007/BF00218191 3555832

[CR34] Lee JH, Asakawa DS, Dennerlein JT, Jindrich DL (2015). Extrinsic and intrinsic index finger muscle attachments in an OpenSim upper-extremity model. Ann Biomed Eng.

[CR35] Maas H, Baan GC, Huijing P (2013). Dissection of a single rat muscle-tendon complex changes joint moments exerted by neighboring muscles: implications for invasive surgical interventions. PLoS ONE.

[CR36] Maas H, Baan GC, Huijing P (2003). The relative position of EDL muscle affects the length of sarcomeres within muscle fibers: experimental results and finite-element modeling. J Biomech Eng.

[CR37] Maas H, Baan GC, Huijing PA (2001). Intermuscular interaction via myofascial force transmission: effects of tibialis anterior and extensor hallucis longus length on force transmission from rat extensor digitorum longus muscle. J Biomech.

[CR38] Maas H, Baan GC, Huijing PA (2004). Muscle force is determined also by muscle relative position: isolated effects. J Biomech.

[CR39] Maas H, Huijing PA (2012). Mechanical effect of rat flexor carpi ulnaris muscle after tendon transfer: does it generate a wrist extension moment?. J Appl Physiol.

[CR40] Maas H, Huijing PA (2012b) Effects of tendon and muscle belly dissection on muscular force transmission following tendon transfer in the rat. J Biomech 45:289–96. doi:10.1016/j.jbiomech.2011.10.026 10.1016/j.jbiomech.2011.10.026 22093795

[CR41] Maas H, Sandercock TG (2010). Force transmission between synergistic skeletal muscles through connective tissue linkages. J Biomed Biotechnol.

[CR42] Maas H, Sandercock TG (2008). Are skeletal muscles independent actuators? Force transmission from soleus muscle in the cat. J Appl Physiol.

[CR43] Purslow PP (2010). Muscle fascia and force transmission. J Bodyw Mov Ther.

[CR44] Raasch CC, Zajac FE, Ma B, Levine WS (1997). Muscle coordination of maximum-speed pedaling. J Biomech.

[CR45] Rack PMH, Westbury DR (1969). The effects of length and stimulus rate on tension in the isometric cat soleus muscle. J Physiol.

[CR46] Ramaswamy KS, Palmer ML, van der Meulen JH (2011). Lateral transmission of force is impaired in skeletal muscles of dystrophic mice and very old rats. J Physiol.

[CR47] Sandercock TG (2000). Nonlinear summation of force in cat soleus muscle results primarily from stretch of the common-elastic elements. J Appl Physiol.

[CR48] Scott SH, Brown IE, Loeb GE (1996). Mechanics of feline soleus: I. Effect of fascicle length and velocity on force output. J Muscle Res Cell Motil.

[CR49] Sharafi B, Blemker SS (2011). A mathematical model of force transmission from intrafascicularly terminating muscle fibers. J Biomech.

[CR50] Siebert T, Till O, Blickhan R (2014). Work partitioning of transversally loaded muscle: experimentation and simulation. Comput Methods Biomech Biomed Eng.

[CR51] Siebert T, Till O, Stutzig N (2014). Muscle force depends on the amount of transversal muscle loading. J Biomech.

[CR52] Silder A, Reeder SB, Thelen DG (2010). The influence of prior hamstring injury on lengthening muscle tissue mechanics. J Biomech.

[CR53] Smeulders MJ, Kreulen M, Hage JJ (2002). Progressive surgical dissection for tendon transposition affects length-force characteristics of rat flexor carpi ulnaris muscle. J Orthop Res.

[CR54] Smeulders MJC, Kreulen M (2007). Myofascial force transmission and tendon transfer for patients suffering from spastic paresis: a review and some new observations. J Electromyogr Kinesiol.

[CR55] Smith LR, Lee KS, Ward SR (2011). Hamstring contractures in children with spastic cerebral palsy result from a stiffer extracellular matrix and increased in vivo sarcomere length. J Physiol.

[CR56] Tian M, Herbert RD, Hoang P (2012). Myofascial force transmission between the human soleus and gastrocnemius muscles during passive knee motion. J Appl Physiol.

[CR57] Tidball JG (1991). Force transmission across muscle cell membranes. J Biomech.

[CR58] Tijs C (2015). Mechanical relevance of linkages that interconnect skeletal muscles.

[CR59] Tijs C, van Dieën JH, Baan GC, Maas H (2014). Three-dimensional ankle moments and nonlinear summation of rat triceps surae muscles. PLoS ONE.

[CR60] Tijs C, van Dieën JH, Maas H (2015). No functionally relevant mechanical effects of epimuscular myofascial connections between rat ankle plantar flexors. J Exp Biol.

[CR61] Tijs C, van Dieën JH, Maas H (2015). Effects of epimuscular myofascial force transmission on sarcomere length of passive muscles in the rat hindlimb. Physiol Rep.

[CR62] Trotter J a, Richmond FJ, Purslow PP (1995) Functional morphology and motor control of series-fibered muscles. In: Exercise and sport sciences reviews, pp 167–213 7556350

[CR63] Whitehead NP, Gregory JE, Morgan DL, Proske U (2001). Passive mechanical properties of the medial gastrocnemius muscle of the cat. J Physiol.

[CR64] Winter SL, Challis JH (2010) The force-length curves of the human rectus femoris and gastrocnemius muscles in vivo. J Appl Biomech 26:45–51 10.1123/jab.26.1.45 20147757

[CR65] Winters J, Woo SL (1990) Hill-based muscle models: a systems engineering perspective. In: Multiple muscle systems. Springer, Berlin, pp 69–93

[CR66] Woittiez RD, Baan GC, Huijing P, Rozendal RH (1985). Functional characteristics of the calf muscles of the rat. J Morphol.

[CR67] Yaman A, Ozturk C, Huijing PA, Yucesoy CA (2013). Magnetic resonance imaging assessment of mechanical interactions between human lower leg muscles in vivo. J Biomech Eng.

[CR68] Yucesoy C, Koopman BHFJM, Grootenboer HJ (2007). Finite element modeling of aponeurotomy: altered intramuscular myofascial force transmission yields complex sarcomere length distributions determining acute effects. Biomech Model Mechanobiol.

[CR69] Yucesoy C, Koopman BHFJM, Huijing P, Grootenboer HJ (2002) Three-dimensional finite element modeling of skeletal muscle using a two-domain approach: linked fiber-matrix mesh model. J Biomech 35:1253–62 10.1016/s0021-9290(02)00069-6 12163314

[CR70] Yucesoy C, Maas H, Koopman BHFJM et al (2006) Mechanisms causing effects of muscle position on proximo-distal muscle force differences in extra-muscular myofascial force transmission. Med Eng Phys 28:214–26. doi:10.1016/j.medengphy.2005.06.004 10.1016/j.medengphy.2005.06.004 16102996

[CR71] Yucesoy CA, Koopman BHFJM, Baan GC (2003). Effects of inter- and extramuscular myofascial force transmission on adjacent synergistic muscles: assessment by experiments and finite-element modeling. J Biomech.

[CR72] Zajac F, Winters J (1990). Modeling musculoskeletal movement systems: joint and body segmental dynamics, musculoskeletal actuation, and neuromuscular control. Mult Muscle Syst.

[CR73] Zhang C, Gao Y (2012). Finite element analysis of mechanics of lateral transmission of force in single muscle fiber. J Biomech.

[CR74] Zhang C, Gao Y (2014). Effects of aging on the lateral transmission of force in rat skeletal muscle. J Biomech.

